# Comparison of clinical outcomes of VISIONAIRE patient-specific instrumentation with conventional instrumentation in total knee arthroplasty: a systematic literature review and meta-analysis

**DOI:** 10.1007/s00402-022-04698-6

**Published:** 2022-11-30

**Authors:** Carsten O. Tibesku, Steven B. Haas, Christopher Saunders, David A. Harwood

**Affiliations:** 1 KniePraxis, Straubing, Germany; 2 Knee Surgery, Hospital for Special Surgery, Weill Cornell Medical College, New York, NY USA; 3 Global Clinical and Medical Affairs, Smith + Nephew, Hull, UK; 4 University Orthopaedic Associates, Somerset, NJ USA

**Keywords:** Total knee arthroplasty, Patient-specific instrumentation, Alignment accuracy, Intraoperative efficiency outcomes, Postoperative outcomes, Return-to-function outcomes, Meta-analysis

## Abstract

**Introduction:**

Malalignment and resulting complications are major challenges in total knee arthroplasty (TKA) which patient-specific instrumentation (PSI) is proposed to alleviate. Previous PSI meta-analyses of TKA outcomes typically do not differentiate between PSI systems and assess relatively few outcomes, so the value of their findings is limited. VISIONAIRE^™^ cutting guides (Smith + Nephew Inc., Memphis, TN, USA) is a PSI system based on preoperative magnetic resonance and X-ray imaging. A systematic literature review (SLR) and meta-analysis, focussed specifically on VISIONAIRE, were conducted to assess TKA accuracy, intraoperative outcomes, and postoperative outcomes, compared with conventional instrumentation (CI).

**Materials and methods:**

The SLR was performed using PubMed, Embase, and Google Scholar databases to identify relevant studies published until March 2022. Depending on statistical heterogeneity, meta-analyses were performed for outcome measures with fixed effect (*I*^2^ < 50%) or random-effects models (*I*^2^ ≥ 50%). Dichotomous outcomes were reported as odds ratios and continuous outcomes were reported as mean differences. Descriptive analyses were performed for outcomes not amenable to meta-analysis.

**Results:**

Outcomes for VISIONAIRE versus CI were reported in 25 studies. Compared with CI, VISIONAIRE reduced odds of mechanical outliers by 40% (*p* < 0.0001), with no statistically significant differences in odds of overall coronal, sagittal, or rotational plane component outliers. VISIONAIRE improved surgical efficiency (operating room, turnover, and tourniquet times reduced by 7.3% (*p* = 0.02), 42% (*p* = 0.022), and 15.9% (*p* = 0.01), respectively), lowering the odds of blood transfusion by 53% (*p* = 0.01) and shortening patients’ hospital stays (11.1% reduction; *p* < 0.0001). There were no significant differences between groups in incidence of postoperative complications and (descriptively analyzed) return-to-function outcomes.

**Conclusion:**

Options for PSI in TKA differ substantially, and it is important to assess the outcomes of individual systems. The current findings suggest that VISIONAIRE guides can lead to improved alignment accuracy and surgical efficiency compared with CI, without compromising postoperative safety and return-to-function outcomes.

**Supplementary Information:**

The online version contains supplementary material available at 10.1007/s00402-022-04698-6.

## Introduction

Total knee arthroplasty (TKA) is generally considered a successful, cost-effective surgical intervention [1]. A major objective of TKA is achieving a neutral mechanical limb alignment, conventionally defined as being within ± 3 degrees of varus/valgus relative to the mechanical axis [2]. Since its emergence in the 1970s and 1980s, TKA has advanced greatly, with various technologies introduced to improve the surgical procedure as well as implant functioning and longevity (e.g. fixation, implant materials, computer-assisted TKA). Nevertheless, malalignment remains a challenge and has been associated with reduced patient satisfaction and postoperative complications, which may necessitate revision [3–6]. Patient-specific instrumentation (PSI) was developed to address this challenge. With PSI, cutting guides specific to the patient’s knee anatomy are used to assist the surgeon with making bony resections [7]. Use of PSI is proposed to lead to improvements in alignment, surgical efficiency, and postoperative patient outcomes, as compared with conventional instrumentation [8]. A number of meta-analyses have compared PSI to conventional instrumentation in TKA, though results have varied [7–19]. Many meta-analyses have reported no overall difference in alignment accuracy between PSI and conventional instrumentation [7, 8, 12–14, 16, 18]. When improvements with PSI over conventional instrumentation were reported for alignment accuracy, these have often been in global mechanical alignment [19] and femoral component alignment [9, 10, 14, 19], though this may be at the cost of an increased risk of outliers for the tibial component alignment [19].

The observed heterogeneity in findings of meta-analyses may be due to the way they are conducted. Previous meta-analyses have typically pooled together studies involving various PSI systems, ignoring technical disparities that may lead to different outcomes [7–19]. For example, PSI systems vary with respect to the imaging technique used, i.e. magnetic resonance imaging (MRI) or computational tomography (CT), surgical planning and manufacturing. Moreover, these meta-analyses often assess only a few outcome measures, such as mechanical alignment [7, 9–13, 17, 18] or patient functional outcomes [15], which can impede robust comparative analysis by overlooking other relevant differences between conventional instrumentation and PSI.

VISIONAIRE™ Cutting Guides (Smith + Nephew Inc., Memphis, TN, USA) are a commonly used PSI system. MRI and X-ray scans of the patient’s bone are taken to identify anatomical landmarks (e.g. anterior–posterior, surgical epicondylar, posterior condylar axes). Preoperatively, a reconstruction of the patient’s knee anatomy, based on the imaging, with the surgeon’s prescribed alignment strategy and operative preferences, are used to manufacture patient-specific cutting guides. Intraoperatively, the guides assist the surgeon with implant alignment, positioning and resections.

VISIONAIRE cutting guides, being customized to the patient’s anatomy, are thought to reduce complexity and invasiveness, conferring surgical benefits to both patient and surgeon [20–22]. An extensive volume of VISIONAIRE-specific data is available from various TKA studies, allowing for robust analysis across several outcome measures. Thus, a systematic literature review (SLR) and meta-analysis were undertaken to investigate the accuracy, intraoperative outcomes, and postoperative outcomes of TKA using VISIONAIRE guides, in comparison with conventional instrumentation.

## Methodology

### Systematic literature search

The SLR and meta-analysis were conducted according to the PRISMA (Preferred Reporting Items for Systematic Reviews and Meta-Analyses) approach. An electronic search was performed using the Embase, PubMed, and Google Scholar databases to identify all relevant clinical studies that detailed the use of VISIONAIRE guides. There was no restriction on publication date and no additional search filters were applied. The following search terms were used: VISIONAIRE [All fields; searched March 2022]. Google Scholar was searched using the terms: VISIONAIRE total knee arthroplasty. Further relevant articles were identified by reviewing the reference lists of identified studies.

### Screening and data abstraction

The titles and abstracts of studies identified in the search were screened. Studies were included only if they compared the outcomes for TKA performed using VISIONAIRE guides versus the outcomes for TKA performed using conventional instrumentation (the comparator). English-language publications reporting randomized controlled trials and observational comparative studies (both retrospective and prospective) were included. Publications were excluded if they were review articles, letters to the editor, commentaries, theses or conference abstracts, or in a language other than English. Furthermore, studies were excluded if they were non-comparative, did not report TKA performed with the VISIONAIRE PSI system, did not include conventional instrumentation as a comparator, were duplicate studies or had duplicated patient populations reporting the same outcomes, did not have human subjects, or otherwise found not to be relevant.

Studies were further screened by full-text review to assess eligibility. Studies were included if they compared VISIONAIRE PSI-enabled TKA with conventional instrumentation-enabled TKA and reported on one or more of the following outcome measures, divided into three categories:

- Alignment accuracy: mechanical axis outliers; coronal component alignment outliers (overall, femoral, tibial), sagittal component alignment outliers (overall, femoral, tibial [posterior slope]); femoral component rotation outliers.
- Intraoperative/perioperative outcomes (indicative of efficiency): operating room time; operating room turnover time; tourniquet time; incidence of blood transfusion; number of instrument trays.
- Postoperative outcomes: incidence of postoperative complications; patient length of stay; return to function.

Screening and data abstraction were performed by two reviewers, with a third reviewer used to resolve disagreements in eligibility, data extraction or usability of data.

### Statistical and descriptive analyses

All meta-analyses were performed with R statistical programming software (version 3.6.1; R Foundation for Statistical Computing, Austria), using the “meta” package. The *I*^2^ statistic was used to characterize heterogeneity [23]. The fixed-effect model was used where there was no evidence of significant heterogeneity between studies (*I*^2^ statistic < 50%), or a random-effects model when heterogeneity was likely (*I*^2^ statistic ≥ 50%). For dichotomous outcomes, the odds ratio (OR) was reported as the summary statistic. For continuous outcomes, the mean difference was reported.

For the following outcome measures assessed with the SLR, meta-analysis could not be performed: number of trays; operating room turnover time; return to function. However, descriptive analyses were undertaken to compare the impact of VISIONAIRE guides versus conventional instrumentation on these outcomes.

## Results

### Literature search

**Fig. 1:**
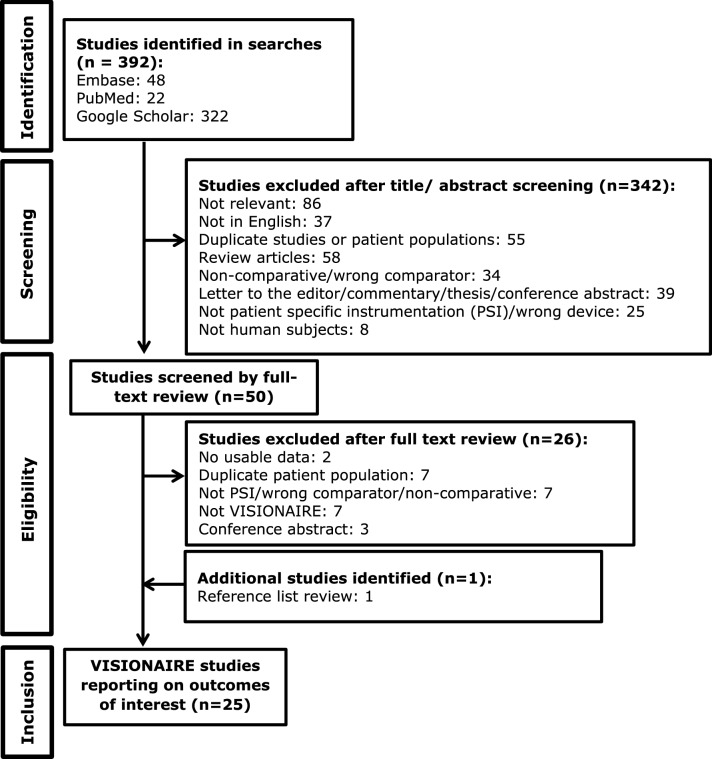
PRISMA diagram for systematic literature review and meta-analysis of outcome measures of interest

The literature search identified 392 studies, with 1 other study identified through reference list review. After screening and eligibility assessment (Fig. 1), 25 relevant studies were identified for inclusion in the meta-analysis (Table 1).

**Table 1:** Study design and size of groups or cohorts receiving TKA enabled with VISIONAIRE guides (treatment) or conventional instrumentation (comparator)

Study	Study design	Knee system	Follow-up time (months)	Group or cohort size VISIONAIRE (number of TKAs)	Conventional Instrumentation (number of TKAs)
Abane et al. [24]	RCT (multicenter)	GENESIS™ II (Smith + Nephew)	3	59	67
Bali et al. [21]	Prospective single-intervention study	Unconstrained cruciate-substituting TKA with GENESIS II implanted with cement	1.5	32 (*32 patients)	10 (*historical in-patient cohort, i.e. 10 of the patients had prior TKA on other side without PSI)
Barke et al. [25]	Retrospective	GENESIS II	N/A; intraop./immediate postop. outcomes assessed	39	50
Broberg et al. [26]	RCT	LEGION	24	25	25
Daniilidis and Tibesku [27]	Retrospective	GENESIS II	3.5	170 (*166 patients)	160 (*160 patients)
DeHaan et al. [28]	Retrospective	Retrospective study of TKAs using LEGION™ or JOURNEY™ (both Smith + Nephew)	N/A; intraop./immediate postop. outcomes assessed	306	50
Huijbregts et al. [29]	RCT	GENESIS II or LEGION	12	69	64
Kosse et al. [30]	RCT	GENESIS II	12	21	21
Marimuthu et al. [31]	Retrospective	LEGION	N/A; alignment outcomes assessed	115	185
Moubarak and Brilhault [32]	Prospective cohort study	GENESIS II or LEGION	N/A; alignment outcomes assessed	57	11
Myers et al. [33]	Retrospective	LEGION or JOURNEY	N/A; intraop./ immediate postop. outcomes assessed	12	19
Nankivell et al. [34]	Prospective (VISIONAIRE group only)	GENESIS II	N/A; intraop. outcomes assessed	41	45 (most recent patients undergoing conventional TKA by same surgeons)
Noble et al. [20]	RCT	LEGION	N/A; alignment outcomes assessed	15	14
Pfitzner et al. [35]	RCT	JOURNEY BCS	3	30	30
Pourgiezis et al. [36]	Prospective (VISIONAIRE group only)	GENESIS II	Alignment assessments: 3–6 months for VISIONAIRE; mean 19 months for conventional instrumentation	45	45 (treated by same surgeons, not prospectively recruited)
Predescu et al. [37]	Observational	GENESIS II	3	40	40
Rathod et al. [38]	Retrospective	LEGION	N/A; intraop./immediate postop. outcomes assessed	30 (*15 patients)	28 (*14 patients)
Stolarczyk et al. [39]	Prospective	Not stated	3	29	29
Stone et al. [40]	Retrospective	LEGION	12	85	85
Tammachote et al. [41]	RCT	GENESIS II	24	51	51
Teeter et al. [42]	RCT	LEGION	24	25	25
Turgeon et al. [43]	RCT	LEGION	24	25	29
Vide et al. [44]	RCT	TC-PLUS™ Total Knee System (Smith + Nephew)	N/A; alignment/intraop. outcomes assessed	47	48
Vundelinckx et al. [45]	RCT	GENESIS II	6	31	31
Zahn et al. [46]	Prospective	TC-PLUS	3	75	150

*BCS* bi-cruciate stabilized; *intraop.* intraoperative; *N/A* not applicable; *postop.* postoperative; *PSI* patient-specific instrumentation; *RCT* randomized controlled trial; *TKA* total knee arthroplasty

*Number of patients (where data are available)

## Outcome measures

### Accuracy

#### Mechanical axis outliers

**Fig. 2:**
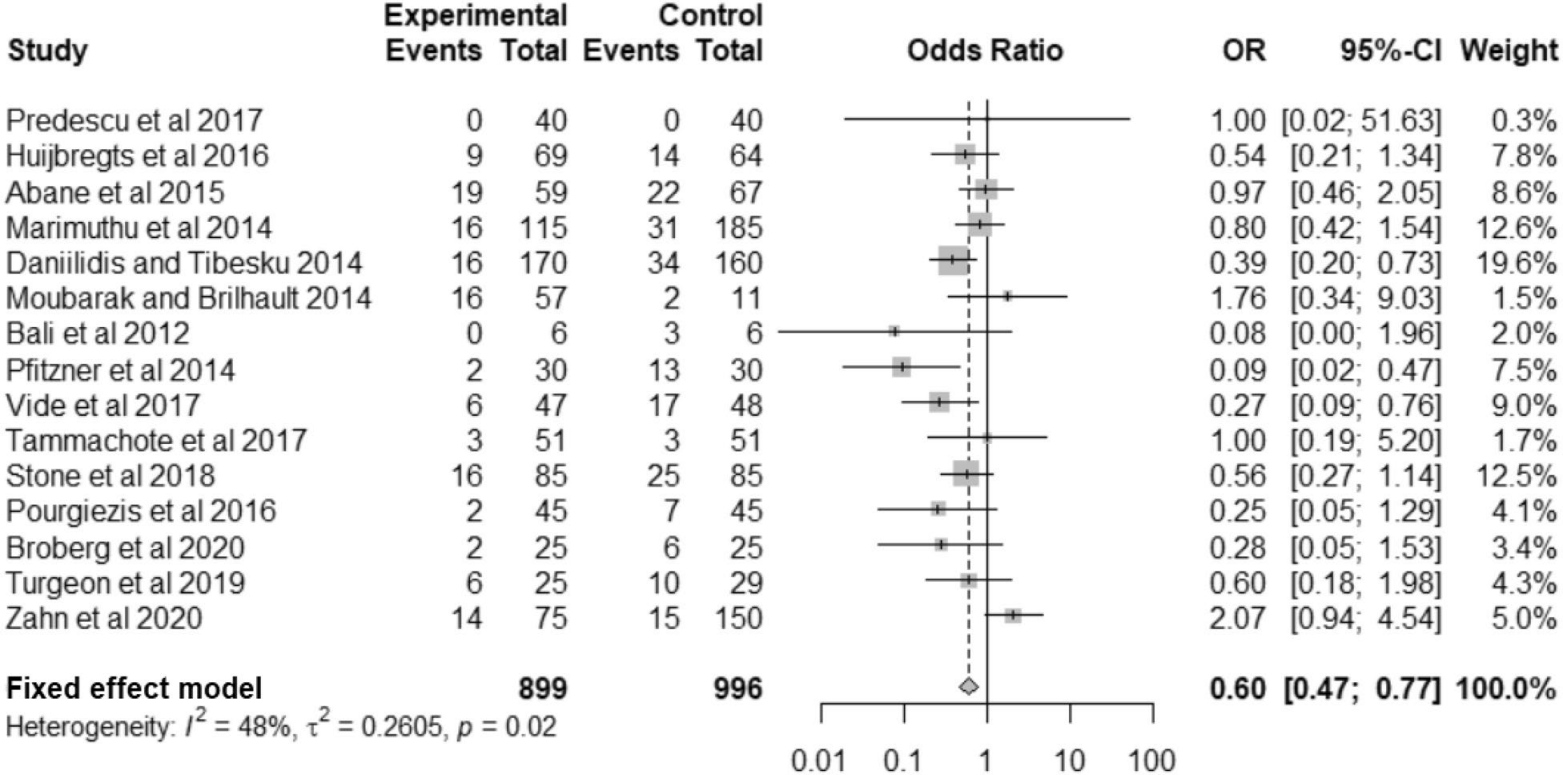
Forest plot for mechanical axis outliers > 3 degrees

Fifteen studies reported number of mechanical axis outliers after TKA [21, 24, 26, 27, 29, 31, 32, 35–37, 40, 41, 43, 44, 46]. Broberg et al*.* (2020) is an updated study of Teeter et al*.* (2019), so they are counted as one study for outliers, with the more recent publication being used for the analysis where possible. Use of VISIONAIRE guides reduced the odds of an outlier in the mechanical axis by 40% compared to conventional instrumentation (OR: 0.60; 95% confidence interval [CI] 0.47–0.77; *p* < 0.0001) (Fig. 2).

Outliers in the coronal plane, sagittal plane (either femoral or tibial components) and rotational component alignment were further investigated (Supplementary Figs. 1–7), and main alignment metrics are presented in Table 2.

**Table 2:** Overall analysis for accuracy outliers

Outcome	Number of studies	VISIONAIRE (number of outliers/total number of measurements)	%	Conventional Instrumentation (number of outliers/total number of measurements)	%	OR	*p* value	Heterogeneity (*I*^2^)
**Mechanical axis**	**15**	**127/899**	**14.1**	**202/996**	**20.3**	**0.60 (0.47–0.77)**	** < 0.0001**	**48%**
**Overall coronal component alignment**	**6**	**55/738**	**7.5**	**71/884**	**8.0**	**0.72 (0.36–1.44)**	**0.35**	**61%**
Coronal femoral component	6	31/369	8.4	39/442	8.8	0.89 (0.54–1.47)	0.65	49%
Coronal tibial component	6	24/369	6.5	32/442	7.2	0.81 (0.46–1.42)	0.46	0%
**Overall sagittal component alignment**	**6**	**135/631**	**21.4**	**114/777**	**14.7**	**1.35 (0.74–2.47)**	**0.33**	**63%**
Femoral flexion (sagittal femoral component)	4	59/288	20.5	43/361	11.9	1.88 (1.20–2.94)	0.0059	42%
Posterior tibial slope (sagittal tibial component)	6	76/343	22.2	71/416	17.1	1.19 (0.64–2.22)	0.57	55%
**Femoral component rotation**	**5**	**34/305**	**11.1**	**56/372**	**15.1**	**0.54 (0.19–1.49)**	**0.23**	**74%**

Rows in bold indicate the main alignment metrics

*OR* odds ratio

#### Outliers in the coronal plane, sagittal plane and rotational component alignment

Meta-analyses of outliers in the coronal plane, sagittal plane and rotational component alignment are shown in Figs. 1–7 of the Supplementary Information section of this publication.

#### Outliers in the coronal plane

Outliers in the coronal plane (either femoral or tibial components) were reported in six studies (Supplementary Fig. 1) [24, 29, 31, 35, 36, 41], and the overall OR was in favour of VISIONAIRE guides, though this was not statistically significant. When femoral component coronal outliers [24, 29, 31, 35, 36, 41] (Supplementary Fig. 2) and tibial component coronal outliers [24, 29, 31, 35, 36, 41] (Supplementary Fig. 3) were assessed separately, the ORs were both in favour of VISIONAIRE guides, but these were not statistically significant.

#### Outliers in the sagittal plane

Outliers in the sagittal plane were reported in six studies [24, 26, 29, 31, 35, 36] (Supplementary Fig. 4). There was no statistically significant difference between the VISIONAIRE group and conventional instrumentation. When the sagittal outliers for femoral components [24, 29, 31, 36] (Supplementary Fig. 5) and tibial components [24, 26, 29, 31, 35, 36] (Supplementary Fig. 6) were assessed separately, there were trends towards more outliers with VISIONAIRE guides, with the former of these being statistically significant.

#### Outliers in femoral component rotation

Femoral component rotation outliers were assessed in five studies [22, 29, 31, 35, 36] (Supplementary Fig. 7). There was a trend towards fewer outliers with VISIONAIRE guides, although this failed to reach statistical significance.

### Intraoperative/perioperative outcomes

#### Operating room time

**Fig. 3:**
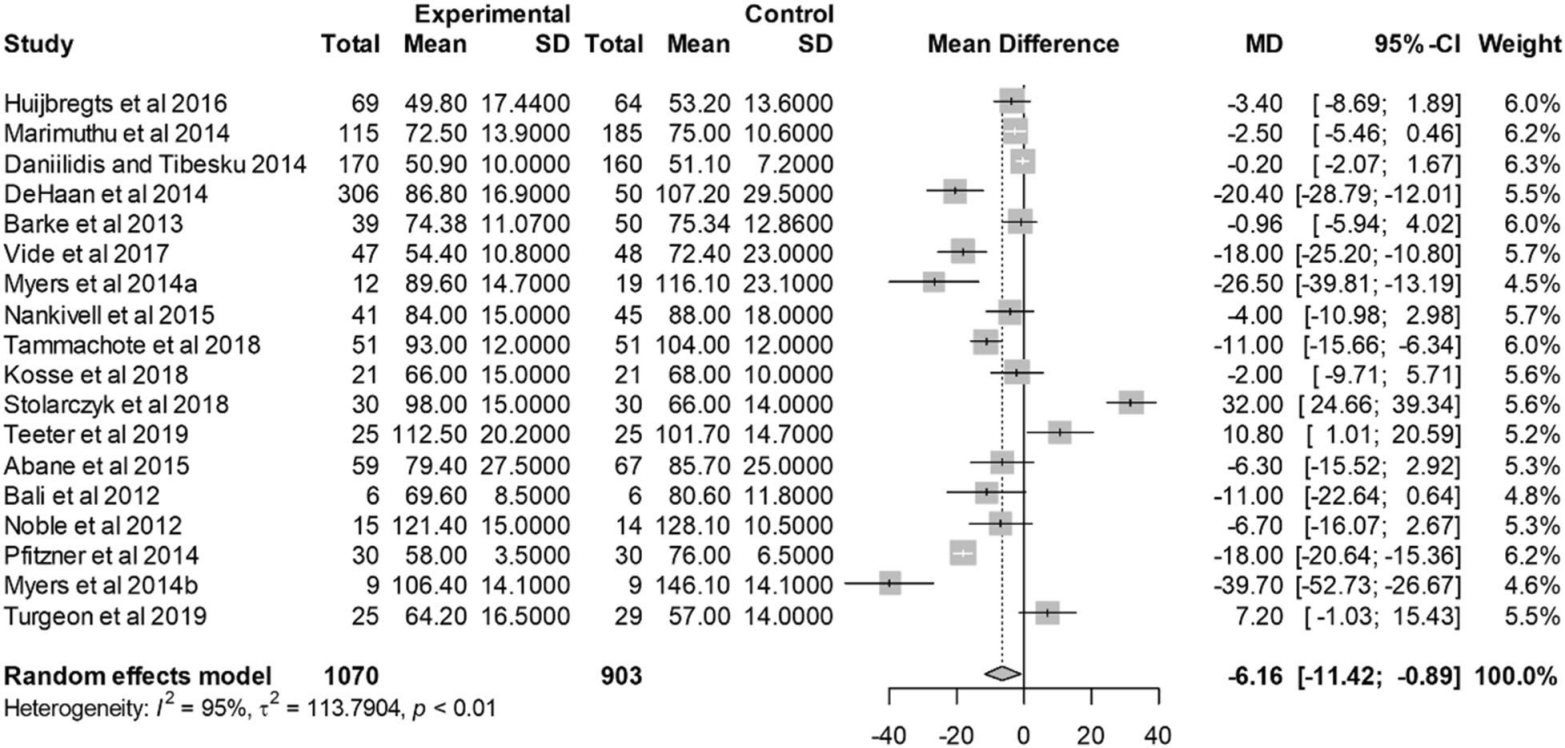
Forest plot for operating room time. **a** Unilateral TKAs (subgroup in study by Myers et al. 2014). **b** Bilateral TKAs (subgroup in study by Myers et al. 2014)

Seventeen studies reported operating room time for TKA with VISIONAIRE guides and conventional instrumentation [20, 21, 24, 25, 27–31, 33–35, 39, 41–44]. There was a significant reduction of time in the operating room with VISIONAIRE guides, compared with conventional instrumentation. The mean difference between VISIONAIRE guides and conventional instrumentation was 6.16 min (95% CI − 0.89; − 11.42; *p* = 0.02; *I*^2^: 95%) (Fig. 3). The weighted mean operating time for conventional TKA was 84.6 min. Using VISIONAIRE guides resulted in 7.3% less operating room time than with conventional instrumentation.

#### Operating room turnover time

One study found that there was a statistically significant difference in operating room turnover time between cases, with VISIONAIRE TKA patients taking 42% less time (6.4 min; *p* = 0.022) [28]. No other studies recorded this metric.

#### Tourniquet time

**Fig. 4:**
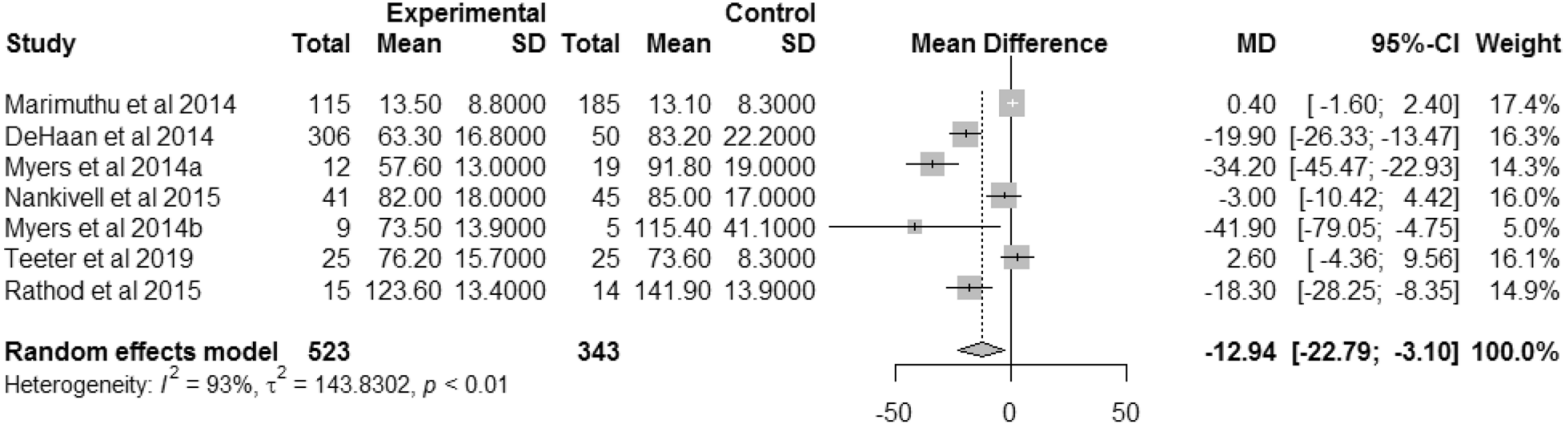
Forest plot for tourniquet time. **a** Unilateral TKAs (subgroup in study by Myers et al. 2014). **b** Bilateral TKAs (subgroup in study by Myers et al. 2014)

Six studies reported tourniquet time [28, 31, 33, 34, 38, 42]. There was a statistically significant reduction in the time needed for patients to have a tourniquet applied. VISIONAIRE techniques required a mean of 12.94 fewer minutes of tourniquet time than conventional instrumentation (95% CI 3.10–22.79; *p* = 0.01; *I*^2^: 93%) (Fig. 4). The weighted mean tourniquet time for conventional TKA was 81.3 min. Using VISIONAIRE guides resulted in 15.9% less tourniquet time than a TKA performed using conventional instrumentation.

#### Blood transfusions

**Fig. 5:**
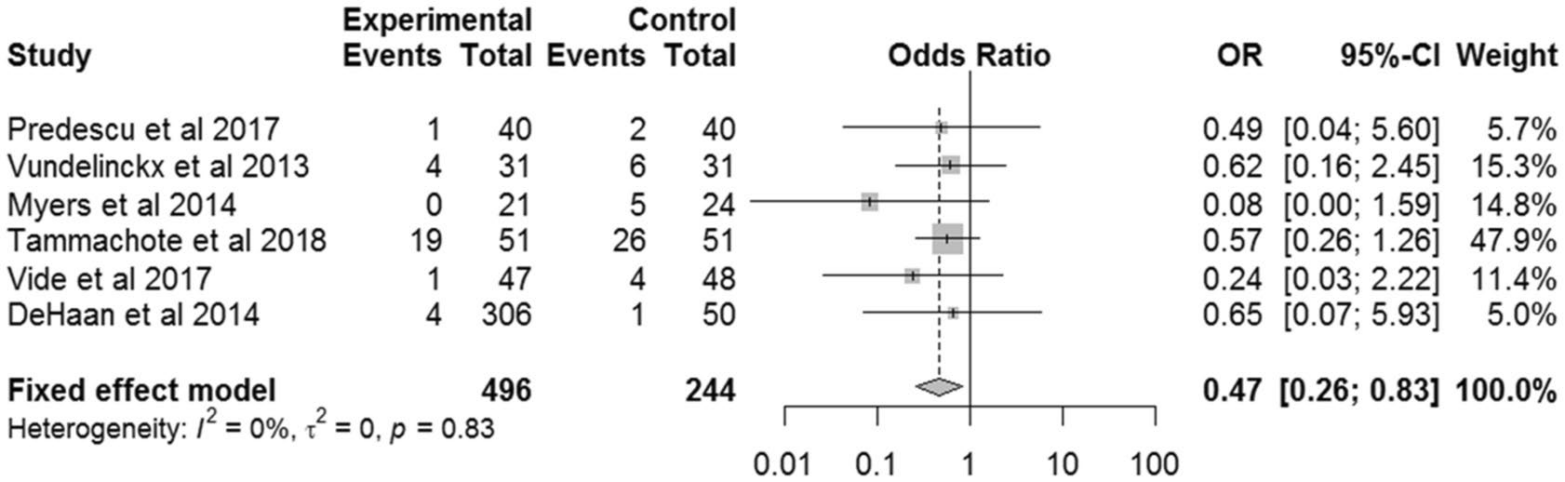
Forest plot for blood transfusions

Six studies reported the odds of blood transfusion with TKA [28, 33, 37, 41, 44, 45]. The odds of patients needing a blood transfusion was 53% lower (OR: 0.47; 95% CI 0.26–0.83) with VISIONAIRE guides compared with conventional instrumentation (*p* = 0.01; *I*^2^ 0%) (Fig. 5). Four of these studies indicated that blood transfusions occurred in the early postoperative period [28, 33, 37, 41], but did not give details on the precise timing of transfusion interventions. Two studies did not specify whether transfusions occurred intraoperatively or postoperatively [44, 45]. Only two studies made specific mention of tranexamic acid usage [37, 41].

#### Number of trays

Six studies reported a reduction in the number of surgical trays (0.6–4.3 fewer trays) when VISIONAIRE guides were used, compared with conventional instrumentation [20, 28, 34, 37, 40, 42]. Dehaan et al. (2014) estimated a cost saving of $240 per case with VISIONAIRE, based on 4 fewer trays (-4 trays x $60 per tray) [28].

### Postoperative outcomes

#### Postoperative complications

**Fig. 6:**
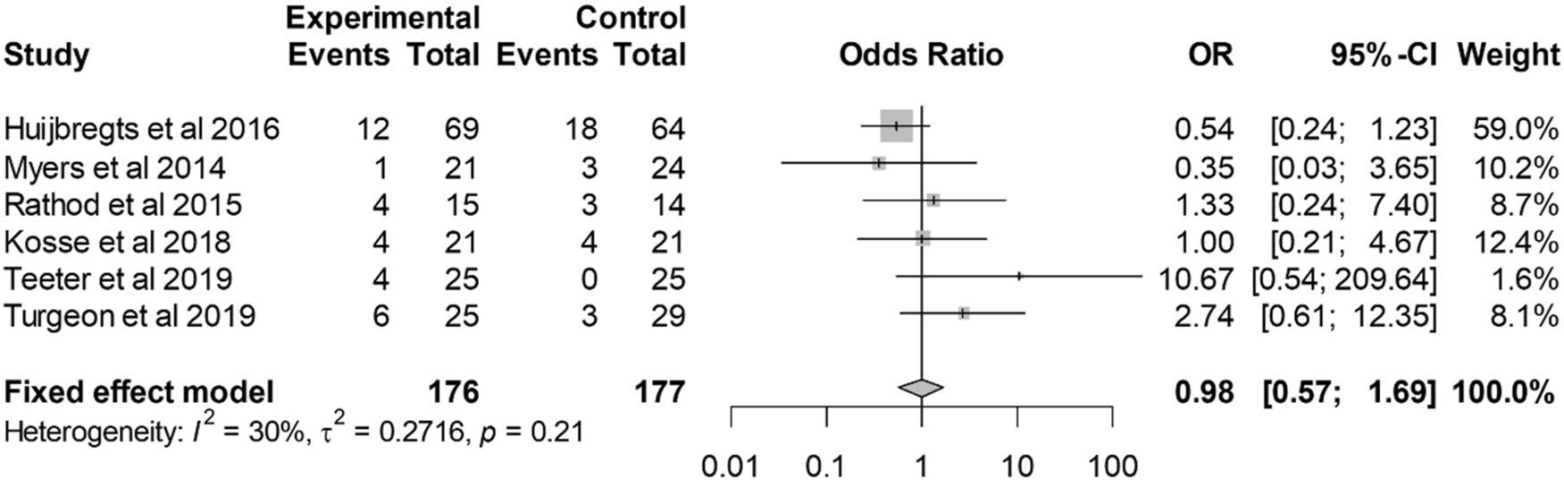
Forest plot for postoperative complications

In the six studies that reported postoperative complications [29, 30, 33, 38, 42, 43], there was no statistically significant difference in the odds of a postoperative complication with VISIONAIRE guides compared with conventional instrumentation (Fig. 6).

#### Length of stay

**Fig. 7:**
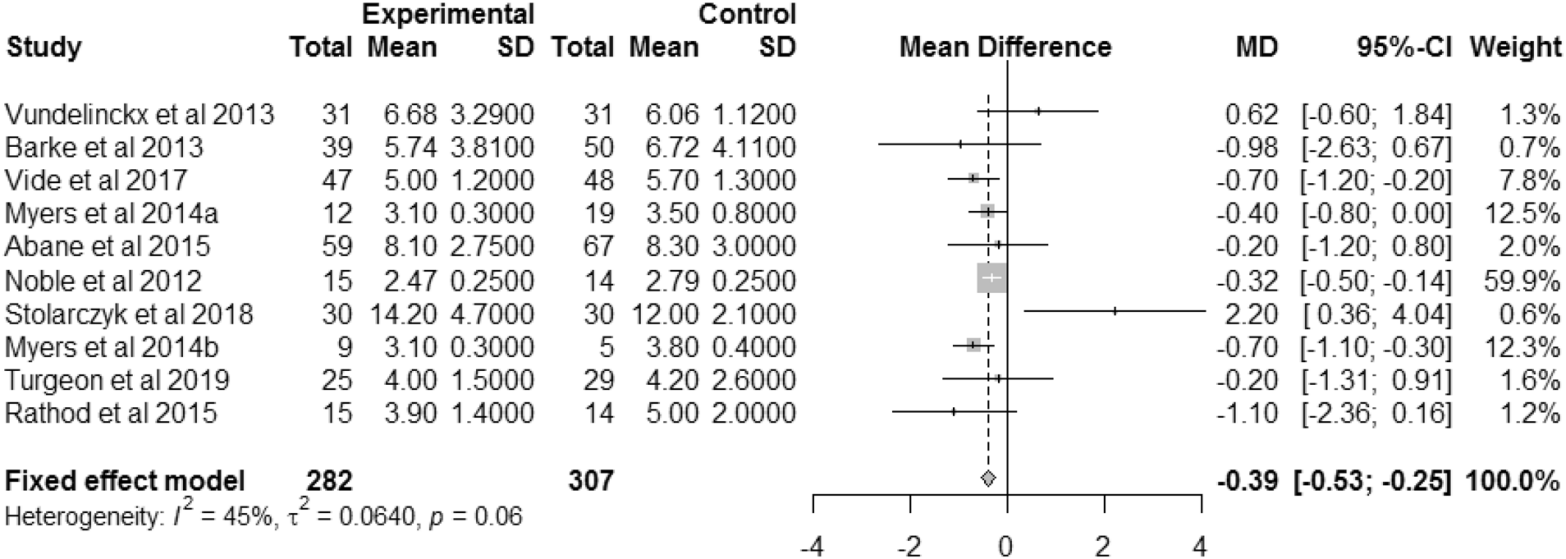
Forest plot for length of stay. **a** Unilateral TKAs (subgroup in study by Myers et al. 2014). **b** Bilateral TKAs (subgroup in study by Myers et al. 2014)

Length of patient stay was reported in nine studies [20, 24, 25, 33, 38, 39, 43–45]. Length of stay was 0.39 days less for surgeries performed with VISIONAIRE guides than with conventional instrumentation (Fig. 7), and the difference was statistically significant (95% CI 0.25–0.53; *p* < 0.0001; *I*^2^: 45%). The weighted mean length of stay for conventional TKA was 3.52 days. This means that patients receiving a TKA with VISIONAIRE guides required 11.1% less time in hospital than patients receiving a TKA performed with conventional instrumentation.

#### Return to function

Eleven studies were identified that included an assessment of postoperative return-to-function, including patient-reported outcome measures and functional outcomes [24, 29, 30, 35, 37, 39–43, 45]. In general, no significant differences were observed between patients who received TKAs enabled with VISIONAIRE guides compared to conventional instrumentation, for various metrics (Table 3). However, in the study by Kosse et al*.* (2018), the VISIONAIRE group scored significantly higher in Knee society score clinical subscale versus conventional instrumentation at 6 weeks (90 vs. 65; *p* = 0.02), though there was no difference at 3 and 12 months, postoperatively [30].

**Table 3:** Return-to-function metrics at final follow-up in all available studies

Study	Follow-up (months)	Joints		Return-to-function metrics at final follow-up	
		VISIONAIRE	Conventional Instrumentation	VISIONAIRE	Conventional instrumentation
Abane et al. [24]	3	59	67	KSS: Knee: 82.5 Function: 82.1 Global: 164.7 OKS: 37.9	KSS: Knee: 83.3 Function: 78.3 Global: 161.7 OKS: 38.2
Huijbregts et al. [29]	12	69	64	*†OKS: 40 SF-12 MCS: 58 SF-12 PCS: 47	*†OKS: 38 SF-12 MCS: 59 SF-12 PCS: 43
Kosse et al. [30]	12	21	21	KOOS: Symptoms: 83 Pain: 94 ADL: 88 Sport: 31 QOL: 69 KSS: Total: 180 Clinical: 96 Function: 80 Kujala: 70 UCLA: 7 VAS pain: 5 VAS satisfaction: 96	KOOS: Symptoms: 75 Pain: 81 ADL: 89 Sport: 58 QOL: 63 KSS: Total: 175 Clinical: 93 Function: 85 Kujala: 62 UCLA: 7 VAS pain: 11 VAS satisfaction: 94
Pfitzner et al. [35]	3	30	30	KSS function: 81 KSS pain: 90 WOMAC: 26	KSS function: 77 KSS pain: 89 WOMAC: 26
Predescu et al. [37]	3	40	40	KSS: 86 KSS function: 88 VAS: 0.9	KSS: 83 KSS function: 85 VAS: 1.0
Stolarczyk et al. [39]	3	29	29	KSS: 74.1 KSS function: 69.3 VAS pain: 2.2	KSS: 69.5 KSS function: 68.2 VAS pain: 2.0
Stone et al. [40]	12	85	85	KSS: 89.4	KSS: 92.0
Tammachote et al. [41]	24	51	51	WOMAC: 5	WOMAC: 4
Teeter et al. [42]	24	25	25	SF-12 MCS: 57.1 SF-12 PCS: 43.0 WOMAC total: 81.6 KSS function: 88.5 EQ-5D: 81.5 UCLA activity: 5.4	SF-12 MCS: 55.4 SF-12 PCS: 45.1 WOMAC total: 82.7 KSS function: 87.9 EQ-5D: 82.5 UCLA activity: 6.1
Turgeon et al. [43]	24	25	29	Improvement in the following outcomes at 2 years: EQ-5D-3L: 0.112 EQ VAS: 9.8 OKS: 13.3 Pain Catastrophizing Scale: 8.0 VAS pain: -40.8 VAS satisfaction: 62.0 UCLA activity: 1.0	Improvement in the following outcomes at 2 years: EQ-5D-3L: 0.120 EQ VAS: 15.0 OKS: 12.8 Pain Catastrophizing Scale: 6.4 VAS pain: -46.3 VAS satisfaction: 56.0 UCLA activity: 1.6
Vundelinckx et al. [45]	6	31	31	KOOS: Total: 311.1 Pain: 70.1 Symptoms: 77.1 ADL: 78.3 Sports: 27.7 QOL: 58.0 Lysholm: 76.7 VAS: 1.4	KOOS: Total: 318.6 Pain: 72.3 Symptoms: 76.8 ADL: 79.7 Sports: 24.2 QOL: 65.6 Lysholm: 81.7 VAS: 1.5

*ADL* activities of daily living; *EQ VAS* EuroQol instrument, visual analogue scale; *EQ-5D* EuroQol instrument, 5 dimensions; *EQ-5D-3L* EuroQol instrument, 5 dimensions, 3 levels; *KOOS* knee injury and osteoarthritis outcome score; *KSS* Knee Society score; *OKS* Oxford Knee score; *QOL* quality of life; *SF-12 MCS* 12-item short form survey, mental component score; *SF-12 PCS* 12-item short form survey, physical component score; *UCLA* University of California Los Angeles scale; *VAS* visual analogue scale; *WOMAC* Western Ontario and McMaster Universities Osteoarthritis Index

*Denotes a statistically significant difference (*p* < 0.05) between groups

^†^A statistically significant difference in OKS was reported between the groups (*p* = 0.049), but this was deemed unlikely to be clinically relevant

## Discussion

In 2008, VISIONAIRE became the first PSI system to receive US Food and Drug Administration clearance [47]. Since then, a plethora of alternative systems has emerged. The effect of technical differences between these systems, such as MRI or CT imaging [8, 19], may be hidden, due to meta-analyses pooling together various PSI systems as a single intervention [8–13, 15, 17, 18]. Only a few previous meta-analyses have separated findings for VISIONAIRE guides from other PSI systems [8, 16, 19]. Thienpont et al*.* (2017) [19] found comparable odds of mechanical malalignment with VISIONAIRE guides versus the Zimmer® PSI system (Zimmer Biomet, USA) (OR: 0.97; *p* = 0.911). VISIONAIRE was shown to be the only PSI system, studied by Lin et al. (2020) [8], that significantly reduced malalignment risk versus conventional instrumentation. Huijbregts et al. (2016) [16] found that VISIONAIRE guides favoured reduced risk of hip-knee-ankle axis outliers versus conventional instrumentation (risk ratio: 0.82), but this was based on only two studies [24, 29]. Although insightful, these meta-analyses focussed on limited outcome measures with respect to VISIONAIRE.

The current SLR and meta-analysis is the first to solely compare VISIONAIRE guides to conventional instrumentation across a range of outcomes. In summary, use of VISIONAIRE guides significantly reduced the odds of a mechanical axis outlier compared to TKA performed with conventional instrumentation. VISIONAIRE guides also led to reductions in operating room, turnover and tourniquet times, and the odds of patients needing a blood transfusion were more than halved. Moreover, with VISIONAIRE guides, patients were able to leave hospital after a significantly reduced stay, and the odds of postoperative complications were roughly equivalent to conventional instrumentation. Furthermore, the balance of evidence suggested TKA using VISIONAIRE guides does not lead to diminished return-to-function outcomes compared with conventional instrumentation.

The standard criterion for accurate alignment, ± 3 degrees from neutral, correlates with functional outcomes and long-term prosthesis survivorship [2, 31, 48]. This meta-analysis observed 40% reduced odds of mechanical axis outliers with VISIONAIRE guides versus conventional instrumentation, consistent with previous meta-analyses that favour PSI systems for alignment accuracy [17, 19]. When considering the overall coronal, sagittal or rotational component, there were no significant differences in the odds of an outlier between VISIONAIRE guides and conventional instrumentation. However, significantly more sagittal outliers for femoral components were observed with VISIONAIRE (OR: 1.88; *p* = 0.0059).

A high demand for surgeries, as well as increasing resource constraints and the operational complexity of this type of intervention, are prompting healthcare systems to improve efficiency within the operating room [49, 50]. This meta-analysis indicates that VISIONAIRE guides are conducive to more efficient TKA procedures. The significantly quicker operating room time, turnover time and tourniquet time observed with VISIONAIRE guides than with conventional instrumentation (by 7.3%, 42%, and 15.9%, respectively) are in line with previous meta-analyses [16, 19]. Improved efficiency is further supported by the observed reduction in surgical trays with VISIONAIRE guides. A reduction in number of trays may be indicative of a quicker, more straightforward operation, improved turnover and cost effectiveness of surgery [28].

Patient blood loss during TKA often necessitates a corrective blood transfusion, but this can lead to clinical and efficiency burdens, due to concomitant complications, prolonged hospital stay and increased surgical complexity [51, 52]. In this meta-analysis, odds of patients requiring a perioperative blood transfusion were reduced by 53% with VISIONAIRE guides compared with conventional instrumentation. This may be related to the fact that TKA performed with PSI systems generally avoid violation of the femoral medullary canal [19]. Included studies sometimes lacked details of the timing of blood transfusions and the full haemostasis protocols used, which may limit interpretation of these findings.

The finding relating to postoperative complications is highly important. It suggests that, despite being a relatively novel surgical technology, VISIONAIRE surgery does not result in greater risk of postoperative complications compared with conventional instrumentation. This may also be reflected in the significantly shorter patient length-of-stay with VISIONAIRE, 11.1% less time in hospital. This finding is consistent with the previous meta-analysis by Huijbregts et al. (2016), which reported a significant difference in favour of PSI for hospital length of stay (8 h shorter) [16].

Increasing emphasis is placed on outcomes of postoperative recovery of function conducive to patient satisfaction [53, 54]. Descriptive analyses of 11 studies suggested no difference in patients’ return-to-function between those having VISIONAIRE-enabled TKAs versus conventional instrumentation. This is in concordance with previous meta-analyses that found non-significant differences in postoperative outcomes between patients receiving TKA with PSI versus conventional instrumentation [15, 16, 19]. In our analysis, only the randomized trial by Kosse et al. (2018) demonstrated a significant difference in postoperative return-to-function between VISIONAIRE guides and conventional instrumentation [30]. A short-term improvement in KSS was seen at 6 weeks, though no significant difference was reported with longer term follow-up [30]. This was attributed to reduced patient-reported pain with VISIONAIRE, and may be due to the absence of intramedullary rods (needed to prepare the femur in conventional TKAs) [30].

The benefits demonstrated with PSI in this analysis should be considered alongside innovative perioperative techniques and new technologies that may also have utility in improving outcomes with TKA, both in terms of implant placement accuracy and subsequent clinical outcomes [55]. For perioperative techniques, numerous facets, such as fast-track strategies, pain management and haemostasis protocols, appropriate patient-profiling, treatment algorithms and patient education, may all contribute further iterative improvements in patient care. Advancements in implant design and assistive technology promise to expand the possibilities for TKA in this constantly evolving field [55]. PSI systems are specifically designed based on the patient’s anatomy to simplify the process of obtaining reliable bony resections and optimal implant alignment; combined with newer more anatomic implant designs, the future of TKA looks to become increasingly personalized with the aim of restoring the patient’s knee joint to its precise pre-disease anatomic and biomechanical status.

In this SLR and meta-analysis, both randomized controlled trials and comparative observational studies were included. Although meta-analyses often assess randomized clinical trials alone, there is an argument that observational studies should not be excluded a priori [56], especially where limited data are available. In surgical disciplines, observational studies provide high external validity, which is important for demonstrating the replication of study outcomes in clinical practice. However, we acknowledge that observational studies may be subject to higher levels of bias. Well-structured, appropriately powered, prospective randomized controlled trials will improve both the quality of the evidence going forward and the internal validity of the findings presented in the current analysis.

Other potential limitations of this meta-analysis include the heterogeneity of surgical techniques performed, differences in the way that outcome measures were reported, and intrinsic limitations of individual studies that may affect the overall quality of evidence. Not all outcomes in studies identified in the SLR were amenable to meta-analysis due to differences in the way they were reported or absence of statistical metrics. To ensure that these outcomes were still evaluated, we performed descriptive analyses. Healthcare economics and value analysis was beyond the scope of this meta-analysis, thus we did not assess the cost-effectiveness of VISIONAIRE guides. However, a recent retrospective real-world study that found that hospital costs associated with VISIONAIRE-enabled TKAs (*n* = 3358) were significantly lower than TKA using conventional instrumentation (*n* = 448,202), $14,910 versus $16,212 (*p* < 0.0001) [47]. As PSI becomes more established in TKA surgeries, it is anticipated that further evidence relating to the cost-effectiveness of VISIONAIRE guides will become available.

## Conclusion

This SLR and meta-analysis indicates that TKA operations utilizing the VISIONAIRE PSI system can be more accurate and efficient than those performed with conventional instrumentation, without compromising safety and patients’ postoperative functional outcomes. Comparison of this meta-analysis with previous meta-analyses suggest that improvements seen with VISIONAIRE guides do not necessarily extrapolate to alternative systems. As patients, surgeons, and payer systems adopt more comprehensive criteria for judging the success of TKA, it will be important to determine the full array of advantages that may be conferred through the use of VISIONAIRE guides.

## Supplementary Information

Below is the link to the electronic supplementary material.

Supplementary file1 (DOC 1837 KB)

## Data Availability

The data used and analyzed for this systematic literature review and meta-analysis are derived from the referenced publications, and are held by Smith + Nephew Inc. The data are available from the authors upon reasonable request, and with the permission of Smith + Nephew Inc.

## Abbreviations

ADL: Activities of daily living
BCS: Bicruciate substituting
CT: Computational tomography
EQ VAS: EuroQol instrument, visual analogue scale
EQ-5D: EuroQol instrument, 5 dimensions
EQ-5D-3L: EuroQol instrument, 5 dimensions, 3 levels
KOOS: Knee injury and osteoarthritis outcome score
KSS: Knee society score
MRI: Magnetic resonance imaging
OKS: Oxford knee score
OR: Odds ratio
PRISMA: Preferred reporting items for systematic reviews and meta-analyses
QOL: Quality of life
SF-12 MCS: 12-Item short form survey, mental component score
SF-12 PCS: 12-Item short form survey, physical component score
PSI: Patient-specific instrumentation
RCT: Randomized controlled trial
SLR: Systematic literature review
TKA: Total knee arthroplasty
UCLA: University of California Los Angeles scale
VAS: Visual analogue scale
WOMAC: Western Ontario and McMaster Universities Osteoarthritis Index
