# Supplementary material for: Comparison of clinical outcomes of VISIONAIRE patient-specific instrumentation with conventional instrumentation in total knee arthroplasty: a systematic literature review and meta-analysis
Source: Arch Orthop Trauma Surg. 2022 Nov 30;143(7):4379–93. doi: 10.1007/s00402-022-04698-6 (PMC10293358; doi:10.1007/s00402-022-04698-6)
Supplement: Supplementary file 1 — Supplementary file1 (DOC 1837 KB) [file 402_2022_4698_MOESM1_ESM.doc]

# **Supplementary information**

**
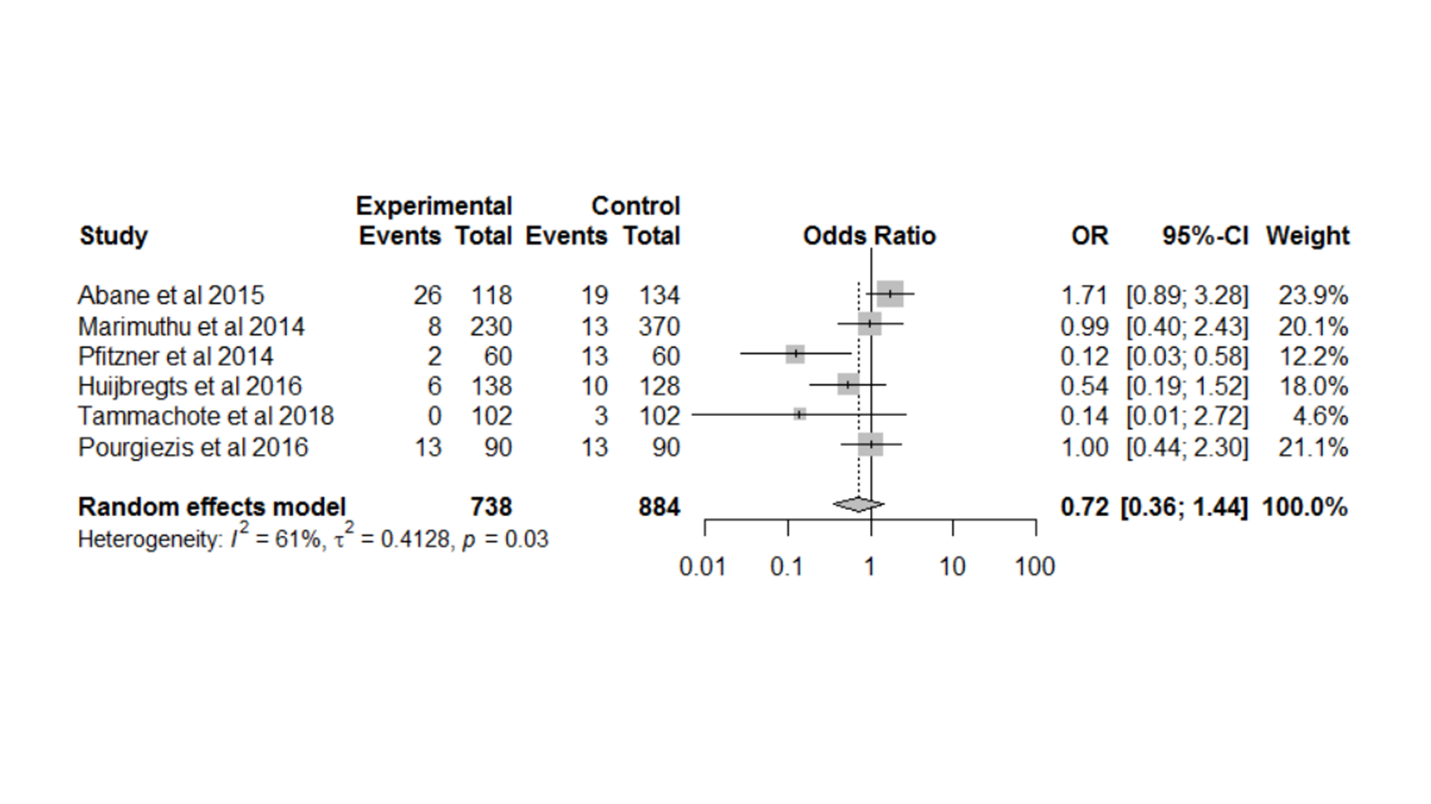
Supplementary Fig. 1** Forest plot of coronal component alignment (both femoral and tibial) outliers >3 degrees

**
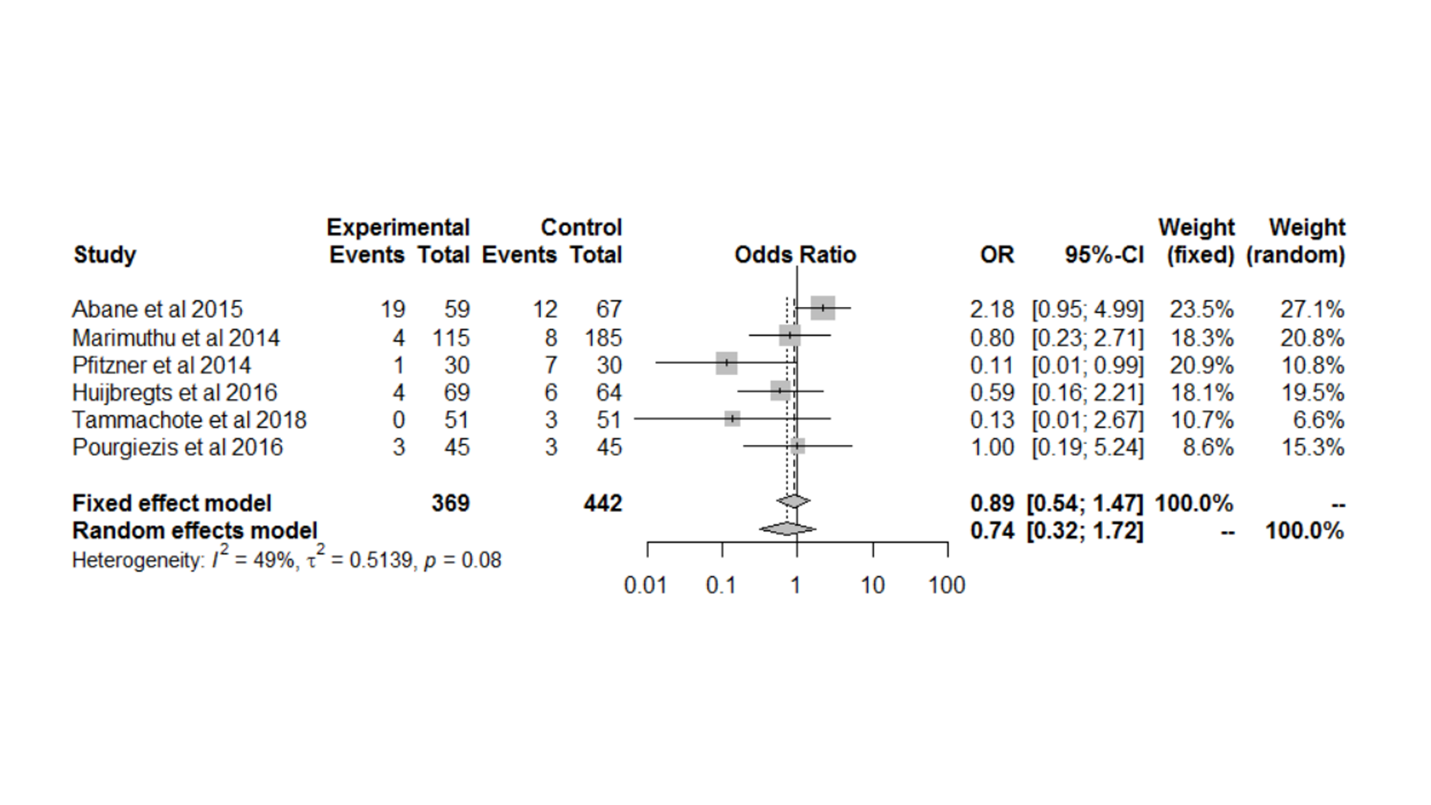
Supplementary Fig. 2** Forest plot for coronal femoral component outliers >3 degrees

**
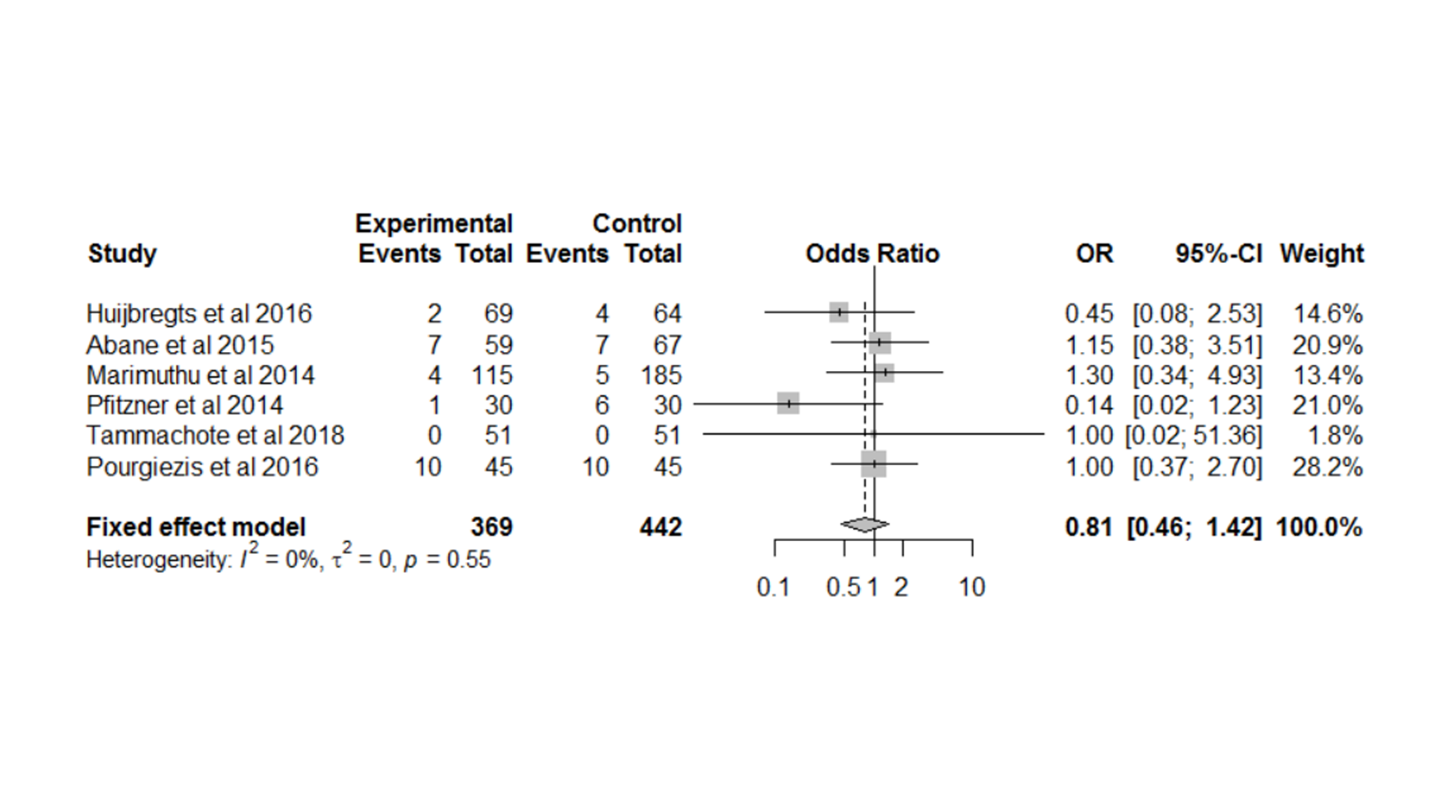
**

**Supplementary Fig. 3** Forest plot for coronal tibial component outliers >3 degrees


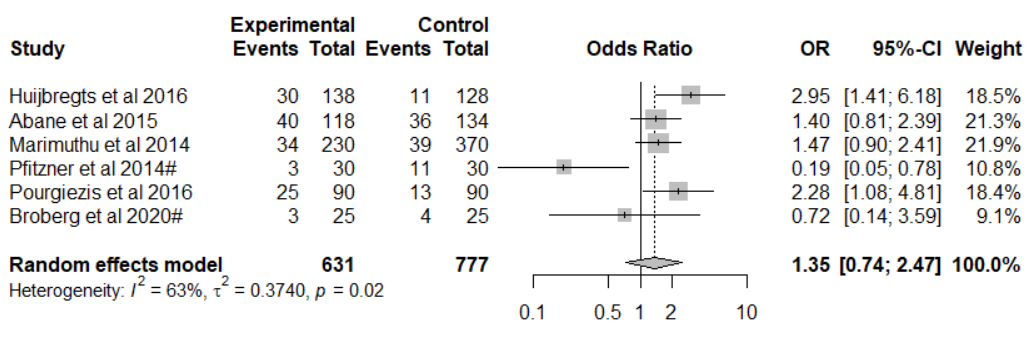


# = study only had data for sagittal tibial component alignment

**Supplementary Fig. 4** Forest plot of sagittal component alignment (both femoral and tibial) outliers >3 degrees

**
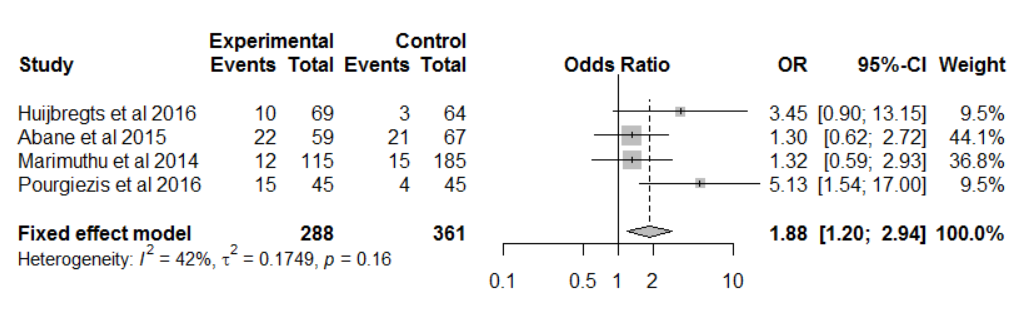
**

**Supplementary Fig. 5** Forest plot of femoral flexion (sagittal femoral component) outliers >3 degrees


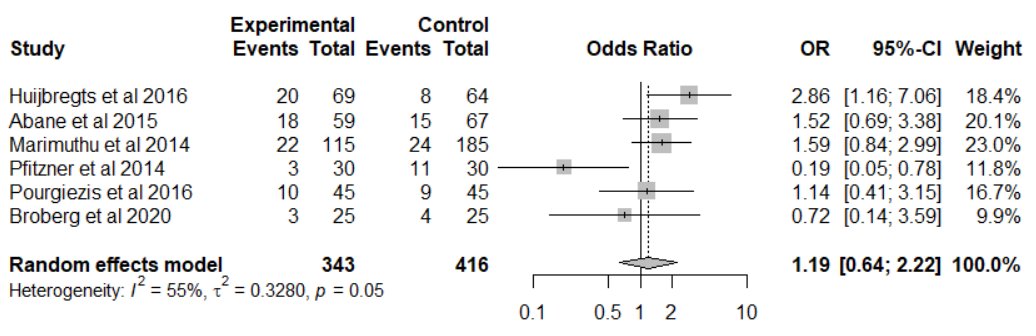


**Supplementary Fig. 6** Forest plot for posterior slope (sagittal tibial component) outliers >3 degrees


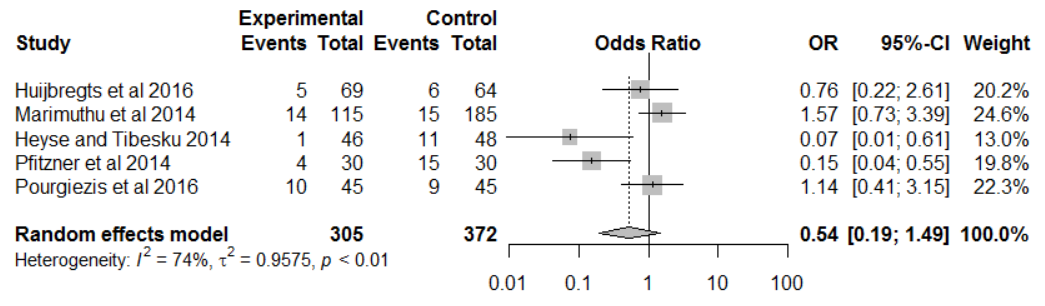


**Supplementary Fig. 7** Forest plot for femoral component rotation outliers >3 degrees
